# Obesity measures in the Kiribati population: a need to reclassify body mass index cut-points

**DOI:** 10.1186/s12889-020-09217-z

**Published:** 2020-07-11

**Authors:** P. E. Eme, B. Burlingame, N. D. Kim, S. Foliaki, C. Wham, J. Douwes

**Affiliations:** 1 Department of Nutrition and Dietetics, University of Nigeria, Nsukka, Enugu State Nigeria; 2 School of Health Sciences, College of Health, Massey University, PO Box 756, Wellington, 6140 New Zealand; 3 Centre of Public Health Research, Massey University, PO Box 756, Wellington, 6140 New Zealand; 4 School of Sport, Exercise and Nutrition, College of Health, Massey University, Private Bag 102904, North Shore City, Auckland, 0745 New Zealand

**Keywords:** Body mass index, Body fat percent, Relationship, Predictor, Adults, Kiribati

## Abstract

**Background:**

Obesity is a public health problem in Micronesia. The objective of the study was to assess obesity, the relationship between body mass index (BMI) and body fat percentage (BF%) among adults, and determine the appropriate BMI cut-points in Kiribati.

**Methods:**

A cross-sectional study was undertaken among 483 adults randomly selected from South Tarawa (ST) and Butaritari (BT). Weight, height, BF% and physical activity level (PAL) was measured using standard methods. Linear and quadratic regression analyses were conducted to assess the association between BF% and BMI whilst controlling for age and gender. Receiver operating characteristics (ROC) curve analyses were used to assess whether for the Kiribati population alternative BMI cut-off points for obesity are needed.

**Results:**

Approximately 75% of participants were obese using standard BMI and BF% cut-offs, with the highest prevalence observed in South Tarawa. BF% was significantly (*p* < 0.001) and positively associated with age (males, *r* = 0.78; females, *r* = 0.67; *p* < 0.001) and BMI. Based on ROC-curve analyses the BMI cut-offs for predicting high BF% among I-Kiribati people were 24.5 kg/m^2^ for males and 32.9 kg/m^2^ for females.

**Conclusions:**

In conclusion, the majority of adults in Kiribati were either obese or overweight and had high BF%. We suggest that ethnic-specific BMI cut-points to define obesity for the population of Kiribati may be more appropriate than the currently used international cut-points.

## Background

The prevalence of overweight and obesity has increased considerably in the past few decades, and has become a significant public health problem globally, with current estimates indicating that 600 million adults are obese and 1.9 billion are overweight [1]. Adult obesity prevalence in Pacific Small Island Developing State (PSIDS), including Nauru (61%), Fiji (30%) and Vanuatu (24%) are among the highest in the world [2]. This is true also for Micronesia, a subregion of Oceania, composed of thousands of small Islands in the western Pacific Ocean, including Kiribati [3]. These Islands mainly rely on the United States for development aid for implementation, acceleration and scaling up of nutrition programmes and policies. However, the sharp increase in obesity rates in this region over the past 40 years shows that current efforts are insufficient to kerb the obesity epidemic [4]. In fact, in 2016, the mean obesity prevalence for adult males and females in Kiribati was 46%, a significant increase from 32.2% in 1997, representing an average annual growth rate of 1.96% [5].

Many studies have shown that obesity, especially central body fatness, is linked with increased risk of morbidity and mortality. In particular, it has been associated with risk factors for coronary heart disease including type 2 diabetes, insulin resistance, and hypertension; cancer; sleep disorders; and anxiety [6, 7]. A significant decrease in physical activity levels and energy expenditure, combined with an increase in energy intake are the main factors contributing to obesity [8].

There are many methods to assess adiposity including measurements of waist circumference, waist-hip ratio, waist-to-height ratio, skinfold calliper measurements, body mass index (BMI), bioelectrical impedance analysis (BIA), under-water weighing (densitometry), near infrared reactance (NIR), magnetic resonance imaging (MRI) and dual-energy X-ray absorptiometry (DEXA) [9]. BMI is inexpensive, relatively easy to calculate and therefore most commonly used, but it does not distinguish between fat and lean body mass. The World Health Organization (WHO) recommends BMI as the most useful population-level measure of overweight and obesity (independent of sex and age), and cut-offs of > 25 kg/m^2^ and > 30 kg/m^2^ are now commonly applied as a definition of overweight and obese, respectively [10]. Body impedance analysis (BIA), a relatively simple, quick, affordable, non-invasive, and reliable body composition method, is widely used to measure percentage body fat (BF%), but is dependent on height and cannot be evaluated independently from fat free mass [11, 12]. The validity of BIA has previously been established for different ethnic groups [13, 14].

Previous studies found a significant positive association between BMI and BF (%) [15–17]. However, the majority of studies were conducted in high-income countries, with only few studies from low-income countries and none from the Pacific region [13, 14]. As a consequence, results from previous studies may not be generalizable to other ethnic populations.

In this study, we aimed to answer the following questions: “what are the adult obesity rates in South Tarawa and Butaritari using different measures?”, and “are the WHO BMI cut-points to defined obesity valid for the I-Kiribati population?” To address these questions we measured, in a sub-population of Pacific Island adults from two atoll Kiribati Islands (one predominantly urban and the other rural), the prevalence of adiposity, and assessed the association between BMI and BF% (using BIA) whilst taking into account age and sex.

## Methods

### Study area

South Tarawa (ST) is the capital of the Republic of Kiribati and is predominantly urban. It is home to about half of the total Kiribati population and most of the government, commercial and education facilities. Butaritari (BT) is the second most northerly of the Gilbert Islands, formerly called Makin Atoll by the US Military, and is rural with a population of 4346 people inhabiting 12 villages [18].

### Participants and design

This was a cross-sectional survey of a household-based sample of adults aged ≥18 years using a multi-stage sampling technique. A total sample of 483 adults (171 from ST and 312 from BT) were recruited using a systematic random sampling method. In particular, in each location, every third house at each site was approached and invited to participate in the study. The inclusion criterion was any household with a mother and father and one or more children living and eating in the same household. All pregnant women and adults who were chronically sick and bedridden were excluded. The respondents were mostly adult (18 years of age) who were household heads and/or those who were involved in the cooking/purchasing of the foods.

Ethical approval was obtained from the Massey University Research Ethics Committee (No: 4000018013). We also obtained a research permit from Kiribati Immigration (RP No- 14/2017). Written consent was obtained from each participant, and data was collected by locally trained research assistants, which took place from August to September, 2018.

### BMI and body fat percentage (BF%)

Height was measured using a height metre and was recorded to the nearest 0.1 cm. Measurements were taken with the subjects bare footed, standing erect with feet parallel, and heels put together in line with methods described by Jellife [19]. Weight (in kg) was measured using a calibrated electronic scale with digital readout (seca 808, Germany) to the nearest 0.1 kg. BMI [weight/height^2^] was used to classify underweight (< 18.0 kg/m^2^), normal (18–24.99 kg/m^2^), overweight (25–29.9 kg/m^2^), obesity class 1 (30–34.99 kg/m^2^), obesity class II (35–39.99 kg/m^2^) and obesity class III (≥40.0 kg/m^2^) [7]. Body composition measurement (corrected for sex, age and height) was carried out using a single bio-impedance analyser system (BC-549, Tanita Corp, Illinois, USA) as per international guidelines [10]. BF% was categorised (low, normal, high, very high) using criteria described by Gallagher et al. [20]. All measurements were taken from 9.00–13.00 h and participants were asked not to engage in vigorous activities 12 h prior to the measurements.

### Physical activity

The short form of the New Zealand Physical Activity Questionnaire (NZPAQ-SF) was used to assess the duration and frequency of brisk walking, and moderate- and vigorous-intensity activities performed in the last 7 days. The NZPAQ-SF, an adaptation of the International Physical Activity Questionnaire (IPAQ), was validated against heart rate monitoring in a multi-ethnic population, including Pacific Islanders, and demonstrated acceptable validity (*r* = 0.25, *p* < 0.001) [21]. Based on frequency (days/week) and average daily duration (min/day) of walking, and moderate and vigorous-intensity activities, metabolic equivalent (MET) values were calculated as follows: METS for walking, moderate- and vigorous-intensity activity (3.3, 4.0, and 8.0, respectively) were multiplied by duration of each activity, summed, and expressed as MET-min/week based on scoring criteria established by the IPAQ Committee for Physical Activity Level (PAL) [22].

### Statistical analysis

All analyses were conducted using SPSS version 20. Linear regression was used to assess associations between BMI and BF%. In addition, we conducted quadratic regression to assess whether the association between BMI and BF% was predominantly linear or curvilinear, similar to other international studies [15, 16]. All regression analyses were controlled for age and stratified by sex (unless indicated otherwise). ROC curve analyses were used to assess whether for the Kiribati population alternative BMI cut-off points for obesity may be needed with improved sensitivity (true positive rate) and specificity (true negative rate). *P-*values ≤0.05 were used to indicate statistical significance.

## Results

Table 1 shows the population characteristics. Weight, BF% and BMI of participants in South Tarawa were significantly (*p* < 0.05) higher than that of participants from Butaritari.

**Table 1:** Population characteristics

	South Tarawa (*N* = 171)	Butaritari (*N* = 312)	Total (*N* = 483)	*P*-value
Age (yrs)(Mean ± SD)	40.8 ± 9.4	40.4 ± 13.6	40.6 ± 12.2	0.740
PAL (Mean ± SD)	1.5 ± 0.2	2.5 ± 1.4	2.2 ± 1.2	*P* < 0.0001
Height (m) (Mean ± SD)	162.0 ± 7.3	161.3 ± 8.2	161.6 ± 7.9	0.374
Weight (kg) (Mean ± SD)	88.6 ± 17.8	78.5 ± 15.2	82.1 ± 16.9	*P* < 0.0001
Body fat, % (Mean ± SD)	38.4 ± 7.8	32.3 ± 11.4	34.5 ± 10.7	*P* < 0.0001
Body mass index, kg/m^2^ (Mean ± SD)	33.7 ± 6.2	30.4 ± 6.6	31.6 ± 6.7	*P* < 0.0001
Males (%)	38 (26.0%)	108 (74.0%)	146 (30.2%)	0.005
Females (%)	133 (39.5%)	204 (60.5%)	337 (69.8%)	
BF% Classification (BIA)				
Low (%)	1 (0.6)	2 (0.6)	3 (1.0)	
Normal (%)	14 (8.2)	110 (35.3)	124 (25.7)	*P* < 0.0001
High (%)	35 (20.4)	104 (33.3)	137 (28.4)	
Very High (%)	121 (70.8)	96 (30.8)	217 (44.9)	
Classification of BMI (kg/m^2^)				
Underweight (%)	1 (0.6)	–	1 (0.2)	
Normal (%)	6 (3.5)	64 (20.5)	70 (14.5)	
Overweight (%)	36 (21.1)	102 (32.7)	138 (28.6)	
Obesity Class I (%)	50 (29.2)	80 (25.6)	130 (26.9)	*P* < 0.0001
Obesity Class II (%)	39 (22.8)	47 (15.1)	86 (17.8)	
Obesity Class III (%)	39 (22.8)	19 (6.1)	58 (12.0)	

The majority (> 70%) of participants in both locations had high BF% and were classified as obese based on BMI results, with again the highest proportion of obesity in South Tarawa.

BMI and age were both consistently and positively associated with BF% for both males and females (Table 2). PAL was inversely associated with BF%, but this was statistically significant only in females, and after controlling for other variables this was no longer significant. In multivariate regression (mutually adjusting for all other variables - age, PAL and location) the regression coefficient (RC) of BMI for males increased from 1.21 to 1.49, but in females it decreased from 0.86 to 0.72.

**Table 2:** Regression models showing the association between BF% (dependent variable) and BMI, age, PAL and location (independent variables) stratified for males and females

	Males				Females			
	Unadjusted regression coefficient (95% CL)	*P*-value	Adjusted^a^ regression coefficient (95% CL)	*P*-value	Unadjusted regression coefficient (95% CL)	*P*-value	Adjusted^a^ regression coefficient (95% CL)	*P*-value
BMI	1.21 (1.06–1.37)	0.000	1.48 (0.45–1.72)	0.000	0.86 (0.79–0.93)	0.000	0.72 (0.60–0.79)	0.000
Age	0.19 (0.07–0.30)	0.000	0.18 (0.10–0.24)	0.040	0.09 (0.03–0.15)	0.000	0.09 (0.04–0.12)	0.000
PAL	−0.89 (−2.49 - -0.71)	0.780	−0.49 (− 0.61- -0.25)	0.680	−0.80 (−1.33 - -0.28)	0.860	− 0.22 (− 0.39 - -0.16)	0.230
Location (ST/BT)	6.43 (3.31–9.54)	0.000	2.04 (1.23–3.21)	0.540	4.30 (2.93–5.66)	0.000	1.92 (1.03–2.61)	0.000
R^2^			0.681				0.683	

*BMI* Body mass index, *PAL* Physical activity level

R^2^: explained variance

^a^Mutually adjusted for all other variables

**Fig. 1:**
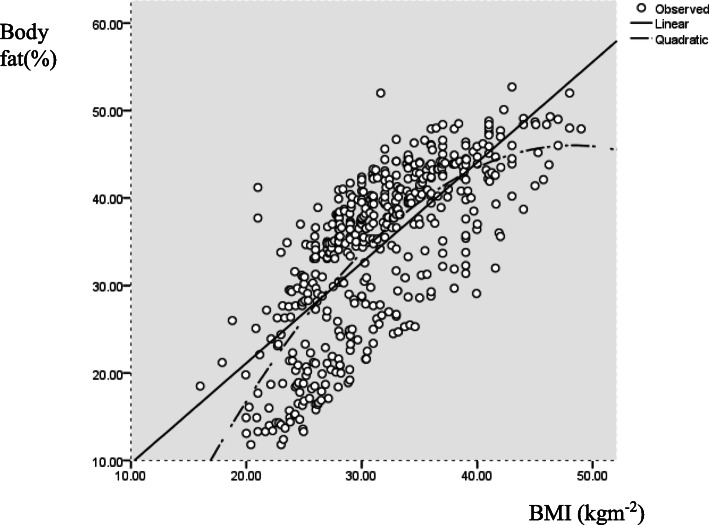
Scatter plot showing linear and quadratic relationship between Body Mass Index (BMI) and body fat percentage (BF%) of I-Kiribati men and women (Linear regression models: BF% male = (BMI × 1.213) + (0.183 × age) - 18.154; BF% female = (BMI × 0.716) + (0.94 × age) + 11.008; Polynomial (quadratic component) regression models: Males (*R2* = 0.614, SEE 5.5%, *p* < 0.000); Females (*R2* = 0.666, SEE 5.4%, *p* < 0.000)

Visual inspection of the scatter plot (Fig. 1) confirmed the positive association between BF% and BMI, which appeared linear in nature and curvilinear towards higher BF% values. Comparing the explained variance between linear and quadratic regression analyses (see Fig. 1) showed only a slight difference i.e. 95% versus 96% in men and 98% versus 98% in women, suggesting that the model fit of the quadratic regression model was not necessarily better than that of linear regression models.

**Fig. 2:**
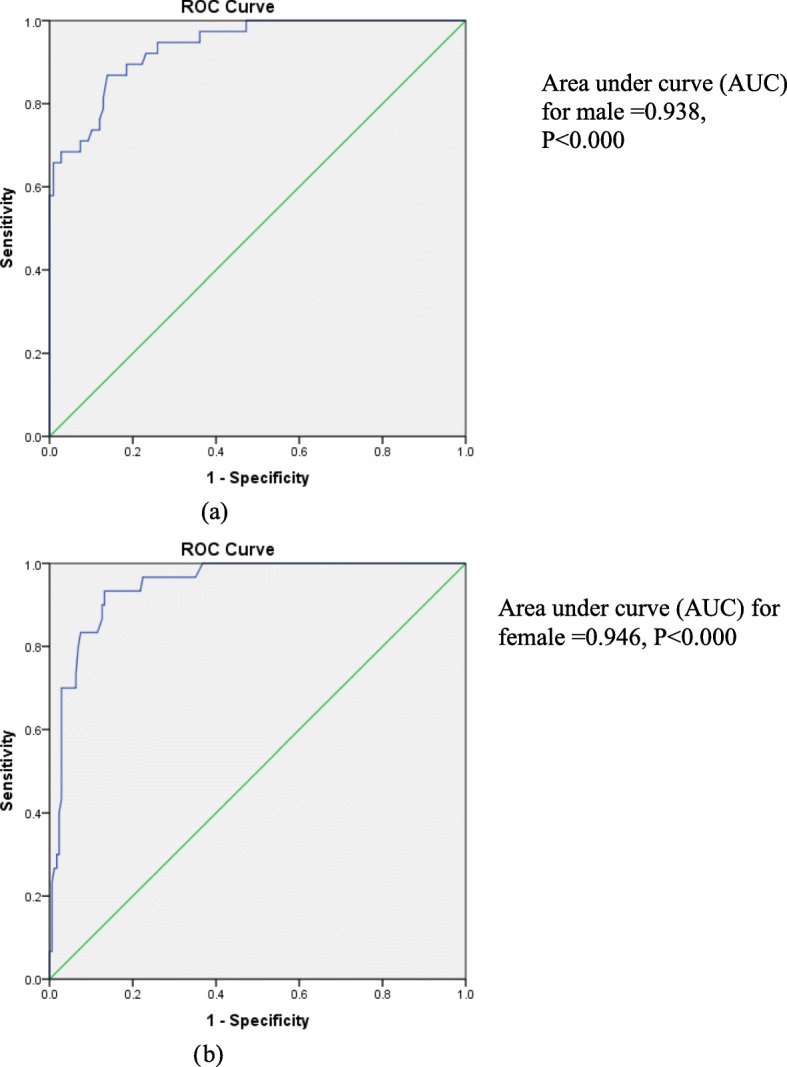
ROC curve in males (**a**) and females (**b**) showing the performance of BMI in predicting BF% in men and women

The area under the ROC curves predict BF% for obesity in men and women based on BMI. The AUCs to predict BF% for obesity reached 0.94 (0.90 to 0.99 with 95% CL) in men, which corresponds to a BMI cut-off value of 24.5 kg/m^2^ (97.4% sensitivity and 64.0% specificity) (*p* < 0.000) (Fig. 2a). In women, the AUC obtained was 0.95 (0.91 to 0.98 95% CL), which correspond to a BMI cut-off value of 32.9 kg/m^2^ (93.3% sensitivity and 86.0% specificity) (*p* < 0.000) (Fig. 2b).

The conventional classification systems were highly specific for both sexes, but their sensitivity was very low for the females. However the proposed classification systems for both sexes had a high specificity and above average specificity.

With the use of the optimal BMI cut-off for the proposed classification system derived from ROC analysis, a truly obese male would be 0.98 times as likely as a truly normal-weight male to be classified as obese, whereas a truly normal-weight male would be only 0.70 times as likely to be classified as obese. For the females, the proposed classification system performed much worse, with positive and negative likelihood ratios of 0.91 and 0.89, respectively as presented in Table 3.

**Table 3:** Sensitivity and specificity for excess fatness for conventional and proposed BMI

	Males		Females	
	Conventional BMI	Proposed BMI	Conventional BMI	Proposed BMI
Sensitivity	0.28	0.64	0.16	0.86
Specificity	0.99	0.97	0.98	0.93
Positive predictive value	0.15	0.98	0.09	0.91
Negative predictive value	0.58	0.70	0.67	0.89

## Discussion

The study was conducted in two atoll Islands that are reasonably representative of the Southern and Northern I-Kiribati population. The prevalence of obesity based on BMI and BF% were 74.8 and 91.2% in ST, and 46.8 and 64.1% in BT, respectively. The mean obesity prevalence using the BMI classification was higher than the national average of 46% reported in 2016 [2], suggesting that the prevalence of obesity in Kiribati may be on the rise. Urgent interventions are therefore needed to curb this increasingly important public health problem.

Our study confirmed a significant positive association between BMI and BF%, which has been demonstrated previously. A study by Rush and colleagues in New Zealand between 1990 and 2004 showed a positive significant relationship between BMI and BF% among Europeans, Maori, Asian adults and Pacific Islanders [23]. Another study by Jackson et al. [24] among Caucasian adults from four clinical centres in US and Canada also showed a significant association between BMI and BF%. Also in agreement with previous studies, our study found that BF% is greater in women [25] and in older age groups [26]. Furthermore, multiple regression analysis showed that sex and age affected the association between BMI and BF%. Therefore, and based on other studies showing similar results [15, 27], this strongly supports that BMI values for predicting BF% need to take into account gender and age (as well as ethnicity as discussed below).

Our study showed the relationship between BMI-BF% was linear in nature but develops curvilinear towards higher BF% values, based on a visual inspection (although a significantly better model fit using quadratic regression was not shown). This is supported by Meeuwsen et al. [16], but differs from results reported by Gallagher et al. [25], which showed a predominantly curvilinear association. Curvilinearity was mainly observed when participants had a BMI of 35 kg/m^2^ or greater indicative of obesity [19]. The same was shown in a study by Jackson et al. [24], which showed that quadratic (curvilinear) effect became most pronounced at BMI levels of ≥35 kg/m^2^. This was also the case for women, and less pronounced in men, in another body composition study from the USA in which half of the subjects had a BMI > 35 kg/m^2^ [26].

The use of BMI cut-off values (based on studies of predominantly European and American Caucasians) to define overweight and obesity for populations with different ethnic backgrounds is controversial. In particular, there are several studies showing that the relationship between BMI and BF% differs among ethnic groups; for example, studies with Indian [27], Indonesian [27], Tongan [28], Australian [29], Jamaican [30] and UK [31] populations have established that BMI represents different values of fat percentage for different populations. This is likely due to differences in energy balance and body build between ethnic groups [23, 27]. The present study showed the optimal cut-off points for predicting high BF% among I-Kiribati people were 24.5 kg/m^2^ for men and 32.9 kg/m^2^ for women. These values vary considerably from the BMI cut-off value derived from American and European Caucasian populations which is 30 kg/m^2^ for both genders [30]. Applying international BMI cut-points in Kiribati (and other countries in the Pacific) may therefore lead to severe misclassification, which may have significant public health implications, and this is why BMI cut-off points for obesity need to be population-specific [32]. In particular, if Kiribati-specific BMI cut-off were used, based on the results of this study, the prevalence estimates of obesity in Kiribati would be considerably greater than current estimate (which is already very high i.e. 46% [7], further emphasising the need for the development of effective public health interventions to reduce the obesity epidemic in Kiribati (and the pacific region more generally). It will also allow more valid comparisons with prevalence estimates of other countries and aid epidemiological research into the causes and mechanisms of obesity and related metabolic conditions in the Pacific region [33].

This study had several limitations. The sample was taken from two different atoll Islands of much disparity in access to health and education facilities and data may therefore not be generalizable to all I-Kiribati. The small population size is another limitation, but being the first of such study in the country and one of only very few in Pacific Island countries, it could serve as a reference. We were unable to control some of the BIA assessment imperatives as we depend on information given by the subjects e.g. despite insurances to the opposite, some may have engaged in vigorous activity in the 12 h prior to when measurement were taken.

## Conclusions

Our results showed that there is high prevalence of obesity in the two atoll Islands of Kiribati using measures of both BMI and BF%. It also demonstrates that BMI is strongly associated with BF% and that this was affected by age and gender. Therefore, our findings support controlling for age and gender when using BMI as a predictor of BF%. Based on our analyses we suggest that ethnic-specific BMI cut-points to define obesity for the population of Kiribati (i.e. 24.5 kg/m^2^ for males and 32.9 kg/m^2^ for females) may be more appropriate than the currently used international cut-points.

## Data Availability

Data cannot be shared because it contains potentially identifying information of the human subjects and will only be shared when the patents related to this research are issued.

## Abbreviations

BMI: Body mass index
BIA: Bioelectrical impedance analysis
NIR: Near infrared reactance
MRI: Magnetic resonance imaging
DEXA: Dual-energy X-ray absorptiometry
WHO: World Health Organisation
BF: Body fat
PSIDS: Pacific Small Island Developing State
PAL: Physical activity level
